# A high-quality reference genome for cabbage obtained with SMRT reveals novel genomic features and evolutionary characteristics

**DOI:** 10.1038/s41598-020-69389-x

**Published:** 2020-07-24

**Authors:** Honghao Lv, Yong Wang, Fengqing Han, Jialei Ji, Zhiyuan Fang, Mu Zhuang, Zhansheng Li, Yangyong Zhang, Limei Yang

**Affiliations:** Institute of Vegetables and Flowers, Chinese Academy of Agricultural Sciences, Key Laboratory of Biology and Genetic Improvement of Horticultural Crops, Ministry of Agriculture, #12 Zhong Guan Cun Nandajie Street, Beijing, 100081 China

**Keywords:** Plant sciences, Genomics

## Abstract

Cabbage (*Brassica oleracea* var. *capitata*) is an important vegetable crop widely grown throughout the world, providing plentiful nutrients and health-promoting substances. To facilitate further genetics and genomic studies and crop improvement, we present here a high-quality reference genome for cabbage*.* We report a de novo genome assembly of the cabbage double-haploid line D134. A combined strategy of single-molecule real-time (SMRT) sequencing, 10× Genomics and chromosome conformation capture (Hi-C) produced a high quality cabbage draft genome. The chromosome-level D134 assembly is 529.92 Mb in size, 135 Mb longer than the current 02-12 reference genome, with scaffold N50 length being raised as high as 38 times. We annotated 44,701 high-quality protein-coding genes, and provided full-length transcripts for 45.59% of the total predicted gene models. Moreover, we identified novel genomic features like underrated TEs, as well as gene families and gene family expansions and contractions during *B. oleracea* evolution. The D134 draft genome is a cabbage reference genome assembled by SMRT long-read sequencing combined with the 10× Genomics and Hi-C technologies for scaffolding. This high-quality cabbage reference genome provides a valuable tool for improvement of Brassica crops.

## Introduction

*Brassica oleracea* is an important vegetable species widely grown throughout the world. The species comprises several subspecies showing extensive morphological and phytochemical diversity. As a diploid species, *B. oleracea* underwent a whole-genome triplication (WGT) event^1^, followed by two whole-genome duplication (WGD) event^2,3^ and a specific WGT^4^ of the Brassiceae lineage, thus becoming a model for studies on polyploid genome evolution. Currently, two *B. oleracea* genomes based on next-generation sequencing (NGS) technology are available: the TO1000 (kale-like; *B. oleracea* var. *alboglabra*) assembly and the 02-12 (cabbage; *B. oleracea* var. *capitata*) assembly^5,6^, but their errors and gaps make them difficult to use for many studies^7–10^. Recently, *B*. *oleracea* L. var. *italica* (broccoli) genome assembly was completed using long reads and optical maps^11^. Broccoli and cabbage belong to Brassica species, however their growth, morphology and molecular levels is extremely variable, showing the importance of generating several genome assemblies for different morphotypes of *B*. *oleracea*. Up to now, there is still no high quality, comprehensive assembled cabbage (*B. oleracea* var. *capitata*) genome, which hinders greatly basic genetics and genomics research, as well as crop improvement. Thus generating an accurate cabbage genome assembly is crucial.

To obtain a homozygous genome, the cabbage double-haploid (DH) line D134 was produced by microspore culture. We conducted whole-genome sequencing and de novo assembly for this line using single-molecule real-time (SMRT) cells on a PacBio Sequel platform combined with high-throughput chromosome conformation capture (Hi-C), next generation sequencing (NGS) and 10× Genomics technologies. This genome assembly is a draft genome for cabbage by Third-Generation Sequencing.

## Materials and methods

### Plant materials

A set of DH cabbage lines were previously obtained from a cross of the two cabbage inbred lines 96–100 and 01–20 (the hybrid was named as Zhonggan 18 for commercial use) using microspore culture, and this DH population has been extensively used in recent years to successfully mapped a series of important agronomic trait genes/QTLs for cabbage, including Fusarium wilt resistance, head weight, plant height, maturing period and seed number^12–16^. Moreover, one of these DH lines, D134, with excellent horticultural characteristics including early maturity, green leaf color and high resistance to Fusarium wilt, has been used constantly in cabbage breeding^17,18^^.^ To better understand its genome background and lay a solid foundation for future cabbage breeding, D134 was chosen for genome sequencing using SMRT technique. High-quality genomic DNA was extracted from fresh leaf tissue using a modified cetyltrimethylammonium bromide (CTAB) protocol^19^, and stored at − 80 °C.

### Genome size estimation and preliminary assembly

We conducted a genomic survey to estimate the genome size, GC content, homozygosity status and duplication content of D134 using a method based on K-mer distribution^20^. A short-insert-size (350 bp) library was constructed using a library construction kit (Illumina) and then sequenced on an Illumina HiSeq 2500 platform. The generated ~ 50 × high-quality reads were used to determine the distribution of K-mer values. We obtained a preliminary assembly by ALLPATHS-LG software^21^.

### Genome sequencing and assembly

At least 50 kb genomic DNA was needed for 20-kb-insert-size library construction. The SMRTbell template was prepared following DNA fragmentation, DNA concentration detection, damage repair/end repair, adapter ligation and DNA purification. The DNA library was sequenced on a PacBio Sequel platform.

Read correction was performed using the PBcR wgs8.3rc1 assembly pipeline. Then, the error-corrected reads were aligned following the “overlap-layout-consensus” paradigm and assembled into contigs by using FALCON with the following parameters: seed_coverage = 60, length_cutoff_pr = 4,000, max_diff = 100, max_cov = 100. Finally, contig assembly correction was performed by mapping all the PacBio data against the generated contigs. Quiver algorithm (with default parameters) was used to polish the contig assembly, and Pilon (with default parameters) was used to perform error correction of contigs using the short paired-end reads generated from an Illumina HiSeq platform (with default parameters).

### 10× Genomics sequencing and scaffolding

We used a GemCode Instrument from 10× Genomics to prepare DNA samples and for automated barcoding. Approximately 1 ng of input DNA was used for the gel-bead-in-emulsion (GEM) reaction. Each input DNA fragment was encapsulated into a GEM and labeled with a unique 10× barcode. GEMs underwent isothermal incubation to generate 10×-barcoded amplicons. Differently barcoded amplicons were mixed and sheared into 500 bp to construct an NGS-ready library. The constructed library was finally sequenced on an Illumina HiSeq X Ten system. The linked reads of the 10× Genomics data were aligned to the contigs generated from PacBio data using bowtie2 with parameters (-D 1 -R 1 -N 0 -L 28 -i S,0,2.50 -n-ceil L,0,0.02 -rdg 5,10 -rfg 5,10 -no-unal), and fragScaff (v2-1)^22^ was used to build scaffolds using the barcoded sequencing reads with parameters (-fs1 '-m 3,000 -q 30 -E 30,000 -o 60,000′ -fs2 '-C 5′ -fs3 '-j 1 -u 3′).

### Hi-C library construction and sequencing

Fresh leaves were cut into 2-cm pieces and fixed with 2% formaldehyde. Hi-C library construction was performed as previously described^23^. In brief, genomic DNA was extracted, and the fixed chromatin was digested by DpnII, followed by a fill-in reaction with biotinylated nucleotides and proximity ligation. The DNA was purified and then sheared into ~ 350-bp fragments using a Covaris S220 device. The DNA fragments were subjected to blunt-end repair, A-tailing, Illumina paired-end adapter ligation and PCR amplification. The Hi-C library was sequenced on an Illumina NovaSeq PE150 instrument.

### Pseudomolecule construction

The Hi-C reads were mapped onto the draft assembly with BWA-MEM^24^ with default parameters. The alignment result was then filtered with mapping quality threshold 30, and duplicate and unmapped reads were removed with SAMTOOLS^24^. The scaffolds were breaked by SALSA with Hi-C clean data. Then the Hi-C clean data was aligned to the finally breaked contigs using BWA software with default parameters. Only the read pairs with both reads in the pair aligned to contigs and mapping quality higher than 30 are considered for scaffolding. According to the linkage information and restriction enzyme site, the string graph formulation was used to construct the scaffold graph with Lachesis (CLUSTER_N = 9, CLUSTER_MIN_RE_SITES = 2,300, CLUSTER_MAX_LINK_DENSITY = 9, CLUSTER_NONINFORMATIVE_RATIO = 0, ORDER_MIN_N_RES_IN_TRUNK = 20, ORDER_MIN_N_RES_IN_SHREDS = 15) and adjusted by juicer box^25^. The quality of the Hi-C assembly was assessed with Mummer software^26^ by collinearity analysis between the present assembly and the genome sequence of closely related species.

### Repeat annotation

Repetitive elements in the D134 genome were predicted using a combination of homology prediction and de novo prediction^27^. For homology prediction, we used RepeatMasker and its in-house scripts (RepeatProteinMask) with default parameters to identify homologous repetitive elements based on Repbase^28^. For de novo prediction, we used RepeatMasker (RepeatMasker at https://repeatmasker.org) to identify repetitive elements based on a predicted database preliminarily generated by RepeatModeler (https://www.repeatmasker.org/RepeatModeler.html), RepeatScout (https://www.repeatmasker.org/) and LTR_finder (https://tlife.fudan.edu.cn/ltr_finder/) software and integrated using UCLUST software with default parameters^29^. And then all repeat sequences with lengths > 100 bp and gap ‘N’ less than 5% constituted the raw transposable element (TE) library. A custom library (a combination of Repbase and de novo TE library which was processed by uclust to yield a non-redundant library) was supplied to RepeatMasker for DNA-level repeat identification.

### Gene annotation

We used a combination of several methods for gene prediction, including de novo prediction, homology based prediction and RNA-Seq assisted prediction. For gene predication based on Ab initio, by AUGUSTUS (https://bioinf.uni-greifswald. de/augustus/) and GlimmerHMM (https://ccb.jhu.edu/software/glimmerhmm/) were used in the automated gene prediction pipeline. For homology annotation, genes from sequenced genomes of *Arabidopsis thaliana* (TAIR10), *B. oleracea* (published), *B. rapa*, *Cucumis sativus*, *Carica papaya*, *Solanum lycopersicum* and *Oryza sativa* were downloaded (Supplementary Table S1) and matched using BLAST (https://blast.ncbi.nlm.nih.gov/Blast.cgi) (v2.2.26; E-value ≤ 1e−5) and then the matching proteins were aligned to the homologous genome sequences for accurate spliced alignments with GeneWise (https://www.ebi.ac.uk/~birney/wise2/) software which was used to predict gene structure contained in each protein region. For RNA-seq prediction, RNA-seq reads were aligned to the assembly using Hisat (v2.0.4)/TopHat (v2.0.11) with default parameters to identify exons region and splice positions and used as input for Stringtie (v1.3.3)/Cufflinks (v2.2.1) with default parameters for genome-based transcript assembly^30^. These predicted results were integrated into a non-redundant reference gene set with EVidenceModeler (EVM, https://evidencemodeler.sourceforge.net/) using PASA (Program to Assemble Spliced Alignment) terminal exon support and including masked transposable elements as input into gene prediction.

We generated functional annotation for the cabbage genes by aligning their predicted protein-coding regions to sequences in publicly available protein databases with BLAST (version 2.2.28+) (with a threshold of E-value ≤ 1e−5). The following protein databases were used: SwissProt (https://www.uniprot.org/), TrEMBL (https://www.uniprot.org/), KEGG (https://www.genome.jp/kegg/) and InterPro (https://www.ebi.ac.uk/interpro/).

### ncRNA annotation

ncRNAs include transfer RNAs (tRNAs), ribosomal RNAs (rRNAs), microRNAs (miRNAs) and small nuclear RNAs (snRNAs). The tRNA genes were predicted by tRNAscan-SE with default parameters^31^. rRNAs are highly conserved; therefore, we aligned the D134 genomes with Arabidopsis rRNAs to identify cabbage rRNAs using BLAST (version 2.2.28+). miRNAs and snRNAs were identified using INFERNAL (https://infernal.janelia.org/) to search the RNA families (Rfam) database with default parameters.

### Gene family identification and expansion analysis

We downloaded genome and annotation data for *A. thaliana* (TAIR10), *R. sativus* (GenBank accession number GCF_000801105.1), *B. rapa* (https://brassicadb.org/brad, version 3.0), *B. oleracea* (https://brassicadb.org/brad, version 1.1; ftp://ftp.ensemblgenomes.org, release 38), *C. rubella* (Phytozome.v1.0), *C. sativus* (GenBank accession number GCF_000004075.1), *S. lycopersicum* (ensembl.plant.v32), *Daucus carota* (GenBank accession number GCF_001625215.1), *Populus trichocarpa* (ensembl.plant.v32), *Vitis vinifera* (Phytozome v9.0), *C. papaya* (Phytozome.ASGPBv0.4), *Gossypium raimondii* (GenBank accession number GCF_000327365.1), *O. sativa* (Nipponbare, IRGSP-1.0), *Zea mays* (ensembl.plant.v32), *Hordeum vulgare* (ensembl.plant.v32), and *Brachypodium distachyon* (ensembl.plant.v32). Gene family identification and expansion analysis were performed as previously described. We chose the longest transcript to represent each gene and removed gene models with open reading frames shorter than 150 bp. Gene family clustering was performed using OrthoMCL^32^ based on the set of 44,701 predicted genes of *B. oleracea* (D134) and the protein sets of the thirteen other dicots and five monocots mentioned above. Based on the OrthoMCL clustering results, gene family expansion analysis was performed using CAFE (https://sourceforge.net/projects/cafehahnlab/).

### Phylogenetic analysis and prediction of evolutionary history

We constructed a phylogenetic tree based on a concatenated sequence alignment of 432 single-copy gene families from D134 and the 17 other plant species using RAxML software with the maximum likelihood method^33^. Multiple sequence alignments were constructed with MUSCLE^34^. Divergence times were estimated by PAML MCMCTree^35^. The Markov chain Monte Carlo (MCMC) process was run for 1,000,000 iterations with a sample frequency of 50 after a burn-in period of 500,000 iterations. The default settings were used for other parameters of MCMCTree. Tracer was used to check for convergence. The following constraints were used for time calibrations: (i) the *A. thaliana* and *O. sativa* divergence time (20.4–30.9 Mya); (ii) the *A. thaliana* and *P. trichocarpa* divergence time (107.0–109.0 Mya); (iii) the *B. distachyon* and *P. trichocarpa* divergence time (140.0–200.0 Mya); and (iv) the *S. lycopersicum* and *A. thaliana* divergence time (107.0–125.0 Mya). The time calibrations were obtained from TimeTree (https://www.timetree.org/).

The branch-site model in CODEML from the PAML package^36^ was used to study positive Darwinian selection on single-copy gene families in *B. oleracea* (D134), *B. oleracea* (02-12), *B. oleracea* (TO1000), *B. rapa* and *R. sativus*. The alternative model for episodic evolution (ω > 1) was tested against the null model (neutral evolution, ω ≈ 1) by comparing likelihood ratios to a chi-square distribution (df = 1)^36^.

### Identification of SNPs and indels

Identification of SNPs and indels was performed as previously described^37^. SNPs and indels (the latter with a length < 100 bp) between the D134 and 02-12 genomes were identified with Mummer as follows: (i) The D134 pseudochromosome sequence was mapped to its corresponding 02-12 pseudochromosome with nucmer with the parameters ‘-mumreference -g 1,000 -c 90 -l 40’. (ii) The delta filter was used to filter mapping noise and determine the one-to-one alignment blocks with the parameters ‘-r -q’. Alignments with aligned positions in one genome that were located more than 10 Mb away in another genome were further filtered. The aligned blocks between these two genomes were identified, and blank regions on the chromosomes that were potential low-similarity regions or multiple-aligned regions were filtered. (iii) Then, show-snps was used to obtain SNPs and small indels (< 100 bp). SNPs and indels shared between the D134 and TO1000 genomes were processed with the same method. The genome distributions of SNPs and indels between the D134 and 02-12/TO1000 genomes were also determined.

## Results and discussion

### Genome sequencing and assembly

To obtain a homozygous genome, the cabbage double-haploid (DH) line D134 was produced by microspore culture. We conducted whole-genome sequencing and de novo assembly for this line using SMRT cells on a PacBio Sequel platform combined with Hi-C, next generation sequencing (NGS) and 10X Genomics technologies. The SMRT cells yielded 8.34 million (64.72 Gb in total with an N50 of 13.3 kb and a mean length of 6.7 kb) PacBio single-molecule long reads (Supplementary Table S2–S6), corresponding to 98 × coverage of the 659.83-Mb cabbage genome as estimated by K-mer distribution analysis. The long reads were corrected using the PBcR wgs8.3rc1 assembly pipeline^38^ and preassembled following the “overlap-layout-consensus” paradigm, generating 870 contigs with an N50 size of 3.68 Mb (Table 1). These contigs were corrected and improved by 103.53 Gb of 10× Genomics short-read data. The initial assembly was 575.74 Mb (preliminary assembly1, 87.3% of the estimated genome size) and composed of 757 scaffolds with an N50 of 8.13 Mb (Tables 1, 2; Supplementary Table S2–S6). Assembly assessment with the core eukaryotic genes mapping approach (CEGMA)^39^ identified 97.58% complete genes and 99.19% complete and partial genes; assembly assessment with Benchmarking Universal Single-Copy Orthologs (BUSCO)^40^ identified 96.1% complete and single-copy genes among 1,440 genes, thus indicating the high quality of the D134 assembly.

**Table 1:** Statistics of the D134 assembly.

	D134 final assembly (including unanchored contigs)	10×-genomics improved assembly (preliminary assembly 1)	Hi-C improved assembly (preliminary assembly 2)
Total assembly length(Mb)	574.91	575.74	575.39
Longest scaffold (Mb)	74.84	19.76	71.59
Number of contigs	902	870	883
N50 contig length (Mb)	3.17	3.68	3.30
Number of scaffolds	682	757	695
N50 scaffold length (Mb)	57.73	8.13	56.58
N90 scaffold length (Mb)	48.45	1.20	46.34

**Table 2:** Comparison of basic sequence statistics among D134, 02-12, TO1000 and HDEM.

	D134 (cabbage)	02-12 (cabbage)	TO1000 (kale-like)	HDEM (broccoli)
Sequenced genome size (Mb)	529,919,152	514,430,932	488,954,160	554,977,060
N50 contig length (Mb)	3,591,417	28,316	21,938	9,813,309
N50 scaffold length (Mb)	61,740,326	1,419,759	48,366,697	58,257,932
Maximum size (Mb)	74,838,096	7,482,359	64,984,695	73,711,317
Gaps	329,204	37,382,998	40,561,975	9,959,204
BUSCO (complete)	95.7%	96.3%	95.8%	95.7%

The D134 assembly was further refined using Hi-C (in vivo fixation of chromosomes) libraries (~ 81.70 Gb of read pairs), resulting in an improved scaffold N50 of 56.58 Mb, with the longest scaffold being 71.59 Mb. We anchored and oriented 181 scaffolds onto nine pseudochromosomes (46.3–71.6 Mb in length), allowing the generation of a final chromosome-level assembly of 529.92 Mb (final assembly without unanchored contigs), covering 92.10% of the 575.39-Mb Hi-C improved assembly (preliminary assembly 2) (Tables 1, 2). The pseudomolecules are hereafter referred to as chromosomes and numbered according to the previously published assembly^5,6^.

### Genome annotation

A comprehensive strategy combining de novo-based prediction, homology-based prediction and RNA-seq-based prediction was applied to annotate protein-coding genes in the D134 genome. The D134 genome comprises 44,701 protein-coding genes, with an average coding-sequence length of 1,057 and an average of 4.8 exons per gene (Supplementary Fig. S1, Supplementary Table S7). The majority of the predicted genes (44,097) were supported by the presence of transcripts from RNA-seq and known functional domains/known homologous proteins (Supplementary Fig. S1, S2; Supplementary Table S7, S8). In addition, we identified full-length transcripts for 45.59% (20,103) of the gene models with Pacific Bioscience SMRT long-read isoform sequencing of five different tissues: the root, stem, leaf, flower and silique. A total of 43,842 (98.10%) of cabbage gene models were functionally annotated based on protein databases including the SwissProt, TrEMBL, Kyoto Encyclopedia of Genes and Genomes (KEGG) and InterPro databases (Supplementary Fig. S3; Supplementary Table S9, S10).

Transposable elements (TEs) reportedly play important roles in shaping genome evolution and gene regulatory networks in many species^41^. We identified 56.47% of the D134 genome as repeat regions and 55.25% as TEs, including retrotransposons (39.51%), DNA transposons (12.38%), and unknown elements (2.66%). Previous studies indicated that long-read assemblies would improve the completeness of catalogue and genomic context of TEs^11^. We re-annotated the TEs in the long-read assembly broccoli HDEM, whose percentage was estimated to be 52.8%, making little difference to the D134 TE content. However, the long-read assemblies contained a much higher proportion of TEs than did the previously published short-read genomes of *B. oleracea*, namely, TO1000 (37.20%) and 02-12 (38.80%), and the genome of its close relative *B. rapa* (22.91%) (Supplementary Table S11). These differences are mainly attributed to the estimated relatively higher proportion of DNA transposons (12.38%) and especially long terminal repeat (LTR) retrotransposons (34.41%) in the D134 genome.

### Evolution of the cabbage genome

**Figure 1:**
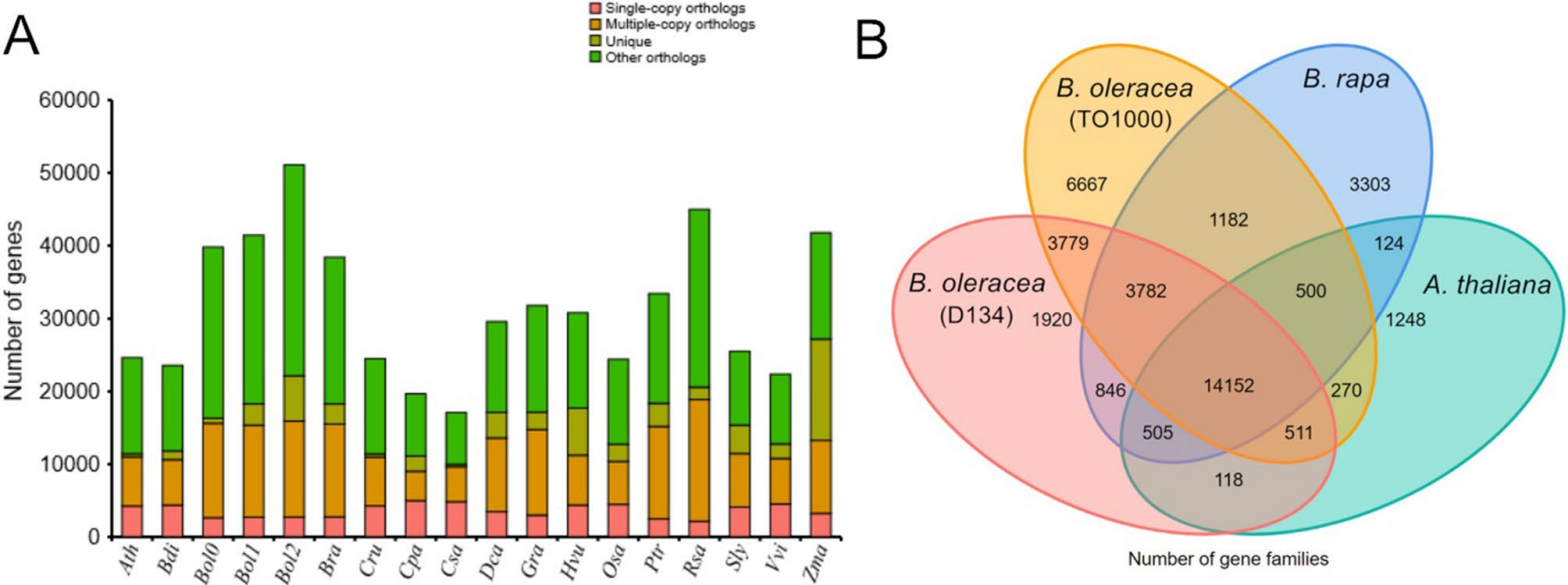
Distribution of genes in cabbage D134 and other representative plant species. (**A**) Orthologous genes found in different plant species. Ath, *A. thaliana*; Bdi, *B. distachyon*; Bol0, *B. oleracea* (D134); Bol1, *B. oleracea* (02-12); Bol2, *B. oleracea* (TO1000); Bra, *B. rapa*; Cru, *C. rubella*; Cpa, *C. papaya*; Csa, *C. sativus*; Dca, *D. carota*; Gra, *G. raimondii*; Hvu, *H. vulgare*; Osa, *O. sativa*; Ptr, *P. trichocarpa*; Rsa, *R. sativus*; Sly, *S. lycopersicum*; Vvi, *V. vinifera*; Zma, *Z. mays*. (**B**) Venn diagram showing unique and shared gene families among *A. thaliana*, *B. rapa* and *B. oleracea* (D134 and TO1000).

**Figure 2:**
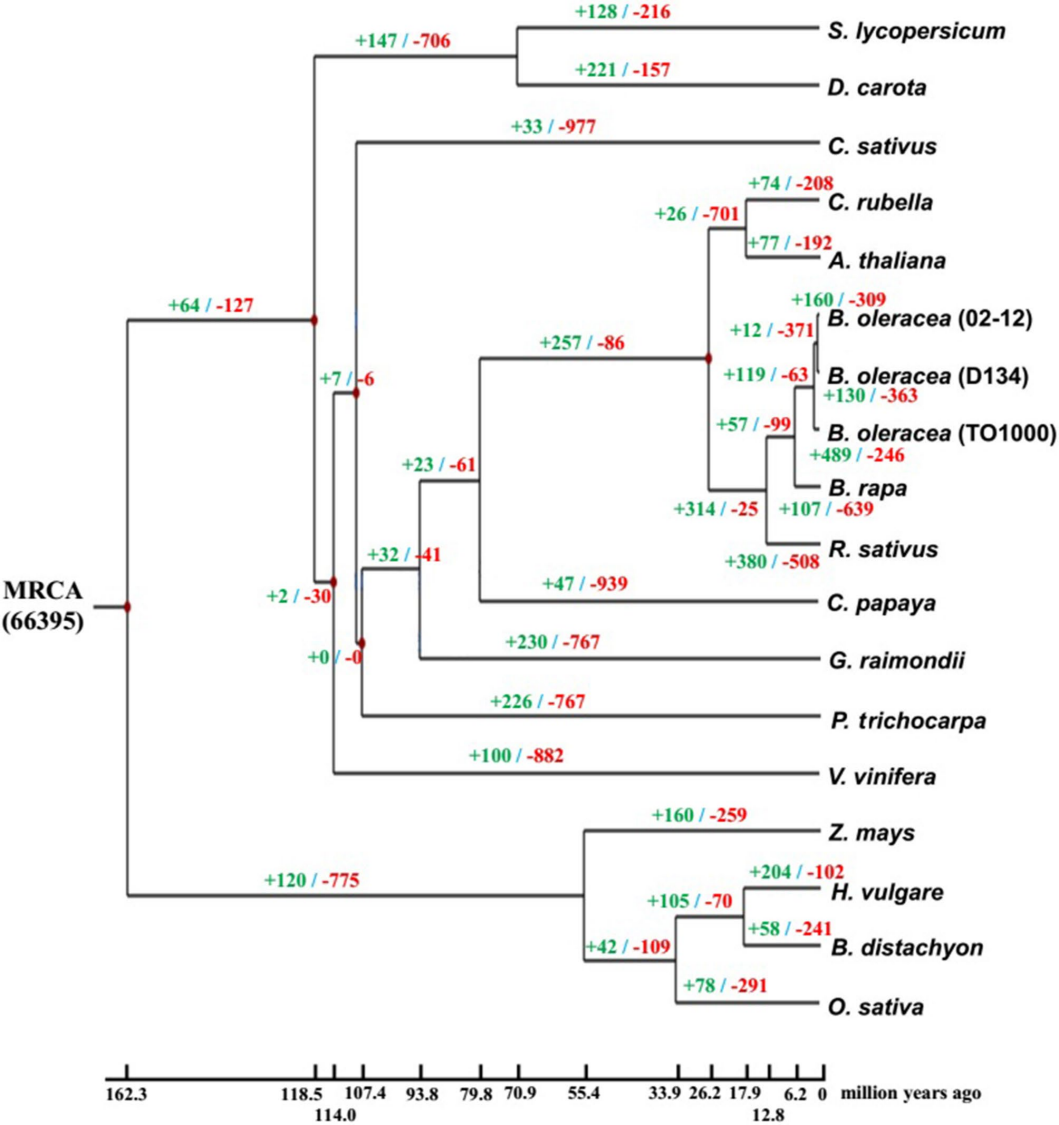
Phylogenetic tree showing divergence times and the evolution of gene family sizes. The phylogenetic tree shows the topology and divergence times for 18 plant species. MRCA, most recent common ancestor. The number in parentheses is the number of gene families in the MRCA as estimated by CAFÉ.

We identified 66,401 gene families from 18 plant species using OrthoMCL (Fig. 1A). Among these gene families, 14,152 were shared by three Brassica species (D134, TO1000, and *B. rapa*) and *Arabidopsis thaliana*, and 1,920 were unique to D134 (Fig. 1B). Using genes extracted from a total of 432 single-copy families, we constructed a high-confidence phylogenetic tree and estimated the divergence times of 18 plant species. As shown in the phylogenetic tree, species of Cruciferae (*B. oleracea* (D134, 02-12, and TO1000), *B. rapa*, *Raphanus sativus*, *Capsella rubella* and *A. thaliana*) were clustered into a specific clade (Fig. 2; Supplementary Fig. S4). The divergence of *C. rubella* and *A. thaliana* from the other four Cruciferae occurred 14.0–24.0 million years ago (Mya), the divergence of *R. sativus* from the other four Brassica species occurred 21.0–30.9 Mya, and *B. rapa* diverged from *B. oleracea* approximately 6.2 Mya (Fig. 2; Supplementary Fig. S4). Moreover, we undertook a computational analysis of gene family sizes to study gene family expansion and contraction during the evolution of *B. oleracea* and related species (Fig. 2). Two hundred and fifty-seven gene families were expanded in the lineage leading to the Cruciferae, whereas 86 families decreased in size (Fig. 2). One hundred and thirty gene families were expanded in D134, compared to 160 in 02-12 and 489 in TO1000 (Fig. 2). Moreover, 363 gene families decreased in size in D134, compared to 309 in 02-12 and 246 in TO1000 (Fig. 2). The expanded gene families in D134 were enriched for protein tyrosine kinase activity, protein metabolic processes, pentose and glucuronate interconversions, and mismatch repair, while the contracted gene families were enriched for transmembrane transporter activity, polycyclic aromatic hydrocarbon degradation, and limonene and pinene degradation (Supplementary Table S12–S15). Compared with 02-12, TO1000, *B. rapa* and *R. sativus*, 157 positively selected genes were identified in D134. These genes were enriched for nuclease activity, noncoding RNA (ncRNA) processing, phenylalanine metabolism, and tyrosine metabolism (Supplementary Table S16, S17).

### Global genome comparison of D134, 02-12 and TO1000

**Figure 3:**
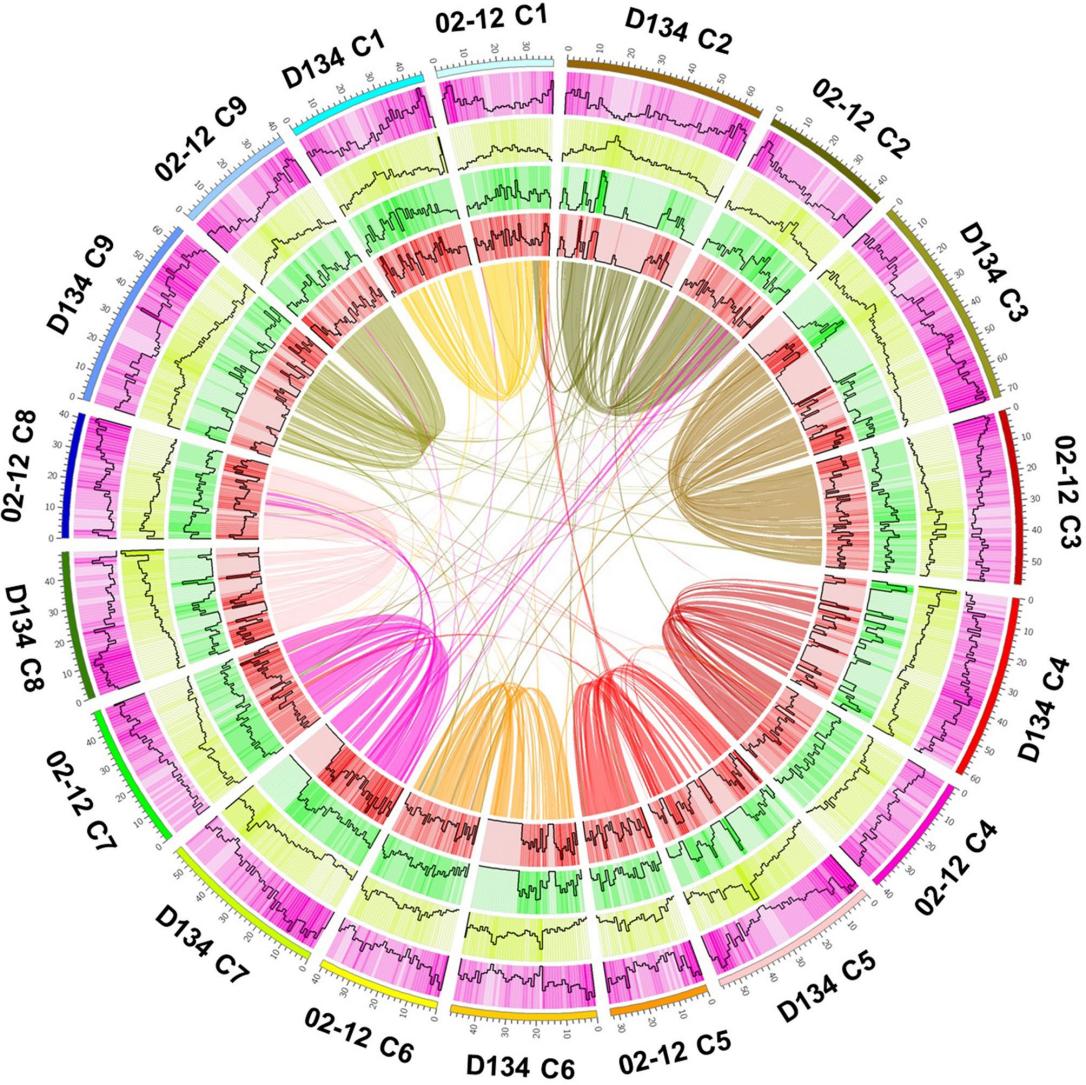
Genomic landscape of D134 and 02-12. Chromosomes, gene density, TE density, SNP density, indel density and best-hit gene pairs are in order from outside to inside in the Circos images.

The 02-12 and TO1000 genome sequences were primarily assembled by whole-genome shotgun sequencing strategies using NGS technologies^5,6^, suggesting that the reference sequences do not cover the entire genome. These two sequenced genomes include numerous gaps, unanchored scaffolds, and even some misordered scaffolds^7^. In this study, the D134 assembly was 135 Mb and 74 Mb longer than the 02-12 and TO1000 reference genomes, respectively (Tables 1, 2). The gaps of D134 assembly are far less than 02-12, TO1000 and HDEM assembly, indicating that the D134 genome was vastly improved with SMRT, Hi-C and 10× Genomics technologies. When the pseudochromosomes of D134 were aligned to the pseudochromosomes of 02-12 and TO1000, approximately 45.78% of the 02-12 genome sequence and 65.19% of the TO1000 genome sequence was matched in one-to-one syntenic blocks with 68.39% and 55.39% of the D134 genome sequences, respectively (Fig. 3, Supplementary Fig. S5; Supplementary Table S18). Moreover, we identified 2,057,052 single nucleotide polymorphisms (SNPs) and 434,689 insertion/deletion polymorphisms (indels) in the syntenic sequences aligned between the D134 and 02-12 genomes and 3,963,977 SNPs and 581,173 indels in those between the D134 and TO1000 genomes (Supplementary Table S19 and S20). The distributions of SNPs and indels were positively correlated (Fig. 3, Supplementary Fig. S5). Most SNPs and indels were distributed in the intergenic regions (Supplementary Table S19 and S20).

## Conclusions

In conclusion, we report a genome assembly of the cabbage double-haploid line D134. A combined strategy of single-molecule sequencing, 10× Genomics and chromosome conformation capture produced a high quality cabbage draft genome. A total of 181 scaffolds accounting for 92.10% of the 575.39-Mb assembly (preliminary assembly 2) were anchored onto nine pseudochromosomes. The D134 assembly is 135 Mb longer than the current cabbage (02-12) reference genome, with scaffold N50 length being raised as high as 38 times. We annotated high-quality protein-coding genes in D134, and provided full-length transcripts for 45.59% of the total predicted gene models (44,701). The D134 displayed dramatic variations and plentiful transposable elements compared with 02-12 and TO1000 reference genomes. Moreover, we identified new gene families and gene family expansions and contractions during *B. oleracea* evolution. This study provides a high-quality cabbage reference genome and facilitates basic research on and improvement of Brassica crops.

## Supplementary information

Supplementary information 1.

## Data Availability

The accession number of genome sequencing data is CNP0000469 in China National GeneBank DataBase (CNGBdb).
