# Supplementary material for: A high-quality reference genome for cabbage obtained with SMRT reveals novel genomic features and evolutionary characteristics
Source: Sci Rep. 2020 Jul 24;10:12394. doi: 10.1038/s41598-020-69389-x (PMC7381634; doi:10.1038/s41598-020-69389-x)
Supplement: Supplementary file 1 — Supplementary information 1. [file 41598_2020_69389_MOESM1_ESM.pdf]

Supplementary information

A high-quality reference genome for cabbage obtained with SMRT reveals novel genomic features and evolutionary characteristics

Honghao Lv[#], Yong Wang[#], Fengqing Han[#], Jialei Ji[#], Zhiyuan Fang, Mu Zhuang, Zhansheng Li, Yangyong Zhang[*] and Limei Yang[*]

Email addresses: lvhonghao@caas.cn; wangyong03@caas.cn; hanfengqing@caas.cn; jijialei@caas.cn; fangzhiyuan@caas.cn; zhuangmu@caas.cn; lizhansheng@caas.cn; zhangyangyong@caas.cn; yanglimei@caas.cn

Institute of Vegetables and Flowers, Chinese Academy of Agricultural Sciences, Key Laboratory of Biology and Genetic Improvement of Horticultural Crops, Ministry of Agriculture, #12 Zhong Guan Cun Nandajie Street, Beijing 100081, China

[#] These authors contributed equally to this work.

[*] Correspondence: yanglimei@caas.cn; zhangyangyong@caas.cn; Tel.: +86-010-621-35629

Table S1. The links of protein sequences from sequenced genomes used for homology based gene prediction.

**Species**	**Download links**
Oryza sativa	ftp://ftp.ensemblgenomes.org/pub/plants/release-32/fasta/oryza_sativa/dna/Oryza_sativa.IRGSP-1.0.dna.toplevel.fa.gz
Solanum lycopersicum	ftp://ftp.ensemblgenomes.org/pub/plants/release-32/fasta/solanum_lycopersicum/dna/Solanum_lycopersicum.SL2.50.dna.toplevel.fa.gz
Arabidopsis thaliana	ftp://ftp.ensemblgenomes.org/pub/plants/release-32/fasta/arabidopsis_thaliana/dna/Arabidopsis_thaliana.TAIR10.dna.toplevel.fa.gz
Brassica rapa	ftp://ftp.ensemblgenomes.org/pub/plants/release-32/fasta/brassica_rapa/dna/Brassica_rapa.IVFCAASv1.dna.toplevel.fa.gz
Carica papaya	http://genome.jgi.doe.gov/pages/dynamicOrganismDownload.jsf?organism=Cpapaya
Cucumis sativus	ftp://ftp.ncbi.nlm.nih.gov/genomes/refseq/plant/Cucumis_sativus/latest_assembly_versions/GCF_000004075.2_ASM407v2
Brassica oleracea	ftp://ftp.ensemblgenomes.org/pub/plants/release-32/fasta/brassica_oleracea/dna/Brassica_oleracea.v2.1.dna.toplevel.fa.gz

Table S2. Summary of the *B.oleracea* genome sequencing data using the PacBio and Illumina technology.

**Pair-end libraries**	**Insert size**	**Total data (G)**	**Read length (bp)**	**Sequence coverage (X)**
Illumina reads	350bp	35.48	150	53.77
Pacbio reads	20Kb	64.72	-	98.09
10X Genomics	-	103.53	150	156.90
Total	-	203.73	-	308.76

Table S3. Sequencing data derived from the PacBio

Read type	Read bases	Read Number	Read length (max)	Read length (mean)	Read length (N50)
Polymerase	56,279,727,290	6,991,696	170,008	8,049	17,827
Pacbio Insertsize	48,049,303,162	6,991,696	170,008	6,872	14,217
Subreads	56,217,049,057	8,337,764	170,008	6,742	13,307

Table S4. The *B.oleracea* genome assembled by Illumina, PacBio and 10X genomics technologies.

	**Contig length(bp)**	**Contig number**	**Scaffold (bp)**	**Scaffold number**
Number ≥ 2000	-	850	-	737
N50	3,679,084	44	8,126,944	24
N60	2,990,971 -	62	5,810,225	33
N70	2,154,286	84	4,233,605	44
N80	1,567,942	115	2,821,861	61
N90	681,583	166	1,197,986	92
Max length	15,061,548	-	19,763,580	-
Total length	574,951,109	870	575,738,899	757

Table S5. Summary of the content of four bases (A, T, G, C) in the assembled *B.oleracea genome*

**Base type**	**Number (bp)**	**Percentage of genome (%)**
A	181,597,927	31.54
T	181,532,057	31.53
C	105,815,972	18.38
G	106,005,153	18.41
N	787,790	0.14
Total	575,738,899	-
GC	211,821,125	36.84

Table S6. Summary of the nine pseudomolecules constructed by Hi-C and comparison of sequence statistics of D134 with 02-12 and TO1000.

Chr	D134 len	02-12 len	TO1000 len	D134 gaps	02-12 gaps	TO1000 gaps
Chr1	46,344,982	38,761,720	43,764,888	25,255	2,714,696	3,885,479
Chr2	66,135,242	44,046,003	52,886,895	45,012	3,151,275	4,908,337
Chr3	71,593,516	57,781,463	64,984,695	32,229	3,621,199	5,341,750
Chr4	61,201,690	40,895,475	53,719,093	35,120	2,685,082	4,997,697
Chr5	56,575,524	32,828,328	46,902,585	56,503	2,150,365	3,976,196
Chr6	48,348,811	48,346,208	39,822,476	20,815	3,074,796	3,675,693
Chr7	56,100,936	40,704,471	48,366,697	40,829	2,868,188	4,315,248
Chr8	49,091,759	41,516,064	41,758,685	13,287	2,670,662	3,358,903
Chr9	65,031,924	40,126,856	54,679,868	60,154	2,710,089	4,885,689
Total	520,424,384	385,006,588	446,885,882	329,204	25,646,352	39,344,992

Table S7. Gene structure annotation in the assembled *B.oleracea genome*

Gene set		Number	Average gene length (bp)	Average CDS length (bp)	Average exons per gene	Average exon length (bp)	Average intron length (bp)
de novo	Augustus	29,907	1,635.34	1,012.09	4.46	226.93	180.13
	GlimmerHMM	76,602	1,234.52	700.42	2.97	236.01	271.42
	SNAP	69,628	1,522.48	731.74	4.07	179.78	257.55
	Geneid	60,185	3,701.51	896.24	4.46	200.97	810.87
	Genscan	44,954	7,086.98	1,194.87	5.79	206.34	1,229.89
Homolog	Arabidopsis thaliana	70,585	1,431.16	915.07	3.07	298.46	249.80
	Cucumis sativus	54,091	1,681.65	1,051.93	3.30	319.24	274.38
	Carica papaya	68,475	1,169.34	774.00	2.66	291.16	238.40
	Solanum lycopersicum	60,991	1,461.57	973.02	2.90	335.70	257.33
	Brassica oleracea_published	47,889	1,754.00	1,037.29	3.31	313.54	310.49
	Brassica rapa	61,586	1,792.03	1,103.10	3.40	324.83	287.54
	Oryza sativa	50,923	1,812.76	1,281.20	3.07	417.62	257.06
RNA-seq	Cufflinks	74,257	3,474.94	1,779.39	6.26	284.16	322.22
	PASA	56,504	2,293.86	1,305.35	5.93	220.20	200.59
EVM		52,503	1,875.83	979.01	4.44	220.35	260.48
ASA-update		60,912	2,002.59	1,098.92	4.99	220.26	226.53
Final set		44,701	2,015.18	1,057.81	4.81	219.70	250.96

Table S8. Gene structure comparison of *B.oleracea genome* and other six sequenced plants.

Species	Number	Average gene length (bp)	Average CDS length (bp)	Average exon length (bp)	Average intron length (bp)	Average exons per gene
Brassica oleracea	44,701	2,015.18	1,057.81	4.81	219.70	250.96
Arabidopsis thaliana	26,869	1,893.81	1,230.33	5.18	237.60	158.80
Cucumis sativus	18,738	3,814.02	1,362.45	5.84	233.21	506.30
Carica papaya	27,751	2,357.42	892.86	4.05	220.20	479.42
Brassica oleracea_published	58,850	1,758.25	1,047.21	4.55	230.00	200.12
Brassica rapa	41,006	2,019.16	1,173.50	5.04	232.95	209.45
Oryza sativa	34,227	2,205.44	1,003.17	3.83	262.06	425.13

Table S9. Gene function annotation of *B.oleracea genome*.

Database		Annotated Num	Annotated Percent(%)
NR		43,823	98.0
Swiss-Prot		34,008	76.1
KEGG		31,819	71.2
InterPro	All	33,474	74.9
	Pfam	32,736	73.2
	GO	23,646	52.9
Annotated		43,842	98.1
Total		44,701	-

Table S10. ncRNA annotation of *B.oleracea genome*.

Type		Copy (w*)	Average length (bp)	Total length (bp)	% of genome
miRNA		1,764	101.15	178,432	0.03
tRNA		1,356	75.18	101,950	0.02
rRNA	rRNA	2,461	360.86	888,081	0.15
	18S	404	1,355.54	547,641	0.09
	28S	1,168	128.71	150,333	0.03
	5.8S	309	387.15	119,630	0.02
	5S	580	121.51	70,477	0.01
snRNA	snRNA	2,453	109.28	268,061	0.05
	CD-box	2,138	105.30	225,142	0.04
	HACA-box	93	129.20	12,016	0.00
	splicing	219	138.88	30,414	0.00

Table S11. Information of the repeat elements in the assembled *B.oleracea genome*

	Denovo+Repbase		TE Proteins		Combined TEs	
	Length (bp)	% in Genome	Length (bp)	% in Genome	Length (bp)	% in Genome
DNA	63,719,816	11.06	26,203,297	4.55	71,316,308	12.38
LINE	19,019,814	3.30	20,217,044	3.51	29,268,060	5.08
SINE	106,379	0.02	0	0.00	106,379	0.02
LTR	194,636,860	33.81	73,687,154	12.80	19,8105,775	34.41
Other	56,488	0.01	0	0.00	56,488	0.01
Unknown	15,300,151	2.66	0	0.00	15,300,151	2.66
Total	304,233,388	52.84	119,902,851	20.82	318,083,209	55.25

Table S12. The GO term enrichment of *B.oleracea* (D134) expanded gene families.

GO ID	GO Term	GO Class	P value	Adjusted P value	x1	x2	n	N	GO level
GO:0004713	protein tyrosine kinase activity	MF	9.12E-29	3.99E-26	62	1357	298	23646	7
GO:0019538	protein metabolic process	BP	1.53E-25	3.34E-23	108	3488	298	23646	4
GO:0044238	primary metabolic process	BP	1.12E-24	1.62E-22	187	8162	298	23646	3
GO:0016310	phosphorylation	BP	1.07E-23	1.17E-21	64	1604	298	23646	6
GO:0006468	protein phosphorylation	BP	1.85E-23	1.62E-21	62	1534	298	23646	7
GO:0071704	organic substance metabolic process	BP	3.03E-23	2.20E-21	188	8407	298	23646	3
GO:0004672	protein kinase activity	MF	6.89E-23	4.30E-21	62	1556	298	23646	6
GO:0043228	non-membrane-bounded organelle	CC	1.75E-22	8.49E-21	42	838	298	23646	3
GO:0043232	intracellular non-membrane-bounded organelle	CC	1.75E-22	8.49E-21	42	838	298	23646	4
GO:0044267	cellular protein metabolic process	BP	3.99E-21	1.74E-19	92	2992	298	23646	5
GO:0043170	macromolecule metabolic process	BP	2.33E-20	8.04E-19	146	6053	298	23646	4
GO:0006464	cellular protein modification process	BP	2.39E-20	8.04E-19	66	1833	298	23646	6
GO:0036211	protein modification process	BP	2.39E-20	8.04E-19	66	1833	298	23646	5
GO:0008152	metabolic process	BP	1.12E-19	3.48E-18	217	11016	298	23646	2
GO:0043412	macromolecule modification	BP	3.86E-19	1.13E-17	66	1894	298	23646	5
GO:0016773	phosphotransferase activity, alcohol group as acceptor	MF	5.05E-18	1.38E-16	62	1776	298	23646	5
GO:0006793	phosphorus metabolic process	BP	6.53E-18	1.59E-16	71	2191	298	23646	4
GO:0006796	phosphate-containing compound metabolic process	BP	6.53E-18	1.59E-16	71	2191	298	23646	5
GO:0016301	kinase activity	MF	1.06E-17	2.43E-16	62	1793	298	23646	5
GO:0032991	macromolecular complex	CC	1.56E-17	3.41E-16	59	1670	298	23646	2

Note: x1: gene number with a GO term in the list; x2: gene number with a GO term in total; n: gene number in the list; N: total gene number.

Table S13. The GO term enrichment of *B.oleracea* (D134) contracted gene families.

GO ID	GO Term	GO Class	P value	Adjusted P value	x1	x2	n	N	GO level
GO:0022804	active transmembrane transporter activity	MF	9.62E-136	3.02E-133	69	360	425	23646	4
GO:0022857	transmembrane transporter activity	MF	4.72E-76	7.40E-74	87	868	425	23646	3
GO:0022891	substrate-specific transmembrane transporter activity	MF	3.01E-66	3.15E-64	69	640	425	23646	4
GO:0005215	transporter activity	MF	2.85E-65	2.23E-63	96	1147	425	23646	2
GO:0022892	substrate-specific transporter activity	MF	1.56E-61	9.78E-60	69	676	425	23646	3
GO:0008324	cation transmembrane transporter activity	MF	7.82E-56	4.09E-54	52	442	425	23646	6
GO:0044765	single-organism transport	BP	4.04E-49	1.81E-47	100	1480	425	23646	3
GO:0016021	integral to membrane	CC	3.69E-45	1.45E-43	85	1193	425	23646	4
GO:0031224	intrinsic to membrane	CC	1.74E-43	6.07E-42	85	1222	425	23646	3
GO:0044425	membrane part	CC	7.52E-43	2.36E-41	97	1531	425	23646	2
GO:0006812	cation transport	BP	1.40E-42	3.99E-41	52	541	425	23646	5
GO:0015075	ion transmembrane transporter activity	MF	1.29E-41	3.36E-40	52	550	425	23646	5
GO:0006811	ion transport	BP	2.13E-39	5.14E-38	56	649	425	23646	4
GO:0005506	iron ion binding	MF	5.36E-37	1.20E-35	47	503	425	23646	7
GO:0020037	heme binding	MF	1.24E-35	2.59E-34	46	498	425	23646	5
GO:0046906	tetrapyrrole binding	MF	1.96E-35	3.84E-34	46	500	425	23646	4
GO:0016705	oxidoreductase activity, acting on paired donors, with incorporation or reduction of molecular oxygen	MF	7.49E-34	1.38E-32	50	595	425	23646	4
GO:0019829	cation-transporting ATPase activity	MF	2.00E-32	3.30E-31	34	112	425	23646	7
GO:0042625	ATPase activity, coupled to transmembrane movement of ions	MF	2.00E-32	3.30E-31	34	112	425	23646	6
GO:0006810	transport	BP	2.66E-31	3.97E-30	110	2228	425	23646	3

Note: x1: gene number with a GO term in the list; x2: gene number with a GO term in total; n: gene number in the list; N: total gene number.

Table S14. The KEGG Pathway enrichment of *B.oleracea* (D134) expanded gene families.

Map ID	Map Title	P value	Adjusted P value	x	y	n	N
map00040	Pentose and glucuronate interconversions	8.11E-27	7.50E-25	37	411	371	20138
map03430	Mismatch repair	2.18E-06	2.37E-05	24	507	371	20138
map03440	Homologous recombination	4.45E-06	4.33E-05	25	553	371	20138
map03030	DNA replication	1.38E-05	0.000106	24	545	371	20138
map03420	Nucleotide excision repair	4.34E-05	0.000297	24	572	371	20138
map00750	Vitamin B6 metabolism	9.79E-05	0.000647	6	41	371	20138
map00196	Photosynthesis-antenna proteins	0.000112	0.000717	6	42	371	20138
map00520	Amino sugar and nucleotide sugar metabolism	0.000592	0.002959	16	366	371	20138
map00906	Carotenoid biosynthesis	0.002375	0.008789	7	99	371	20138
map04660	T cell receptor signaling pathway	0.002848	0.010331	8	130	371	20138
map04144	Endocytosis	0.006982	0.022662	23	707	371	20138
map03010	Ribosome	0.008926	0.027723	26	842	371	20138
map04062	Chemokine signaling pathway	0.009087	0.027723	8	158	371	20138
map00073	Cutin, suberine and wax biosynthesis	0.009141	0.027723	9	191	371	20138

Note: x: gene number with a KEGG term in the list; y: gene number with a KEGG term in total; n: gene number in the list; N: total gene number.

Table S15. The KEGG Pathway enrichment of *B.oleracea* (D134) contracted gene families.

**Map ID**	**Map Title**	**P value**	**Adjusted P value**	**x**	**y**	**n**	**N**
map00624	Polycyclic aromatic hydrocarbon degradation	2.63E-38	1.50E-36	38	126	318	20138
map00903	Limonene and pinene degradation	2.45E-35	9.29E-34	38	148	318	20138
map00627	Aminobenzoate degradation	9.73E-35	2.77E-33	38	153	318	20138
map00945	Stilbenoid, diarylheptanoid and gingerol biosynthesis	1.02E-33	2.33E-32	38	162	318	20138
map00624	Polycyclic aromatic hydrocarbon degradation	2.63E-38	1.50E-36	38	126	318	20138
map00903	Limonene and pinene degradation	2.45E-35	9.29E-34	38	148	318	20138
map00627	Aminobenzoate degradation	9.73E-35	2.77E-33	38	153	318	20138
map00945	Stilbenoid, diarylheptanoid and gingerol biosynthesis	1.02E-33	2.33E-32	38	162	318	20138
map03440	Homologous recombination	7.97E-10	7.57E-09	27	553	318	20138
map00531	Glycosaminoglycan degradation	2.49E-09	2.02E-08	13	93	318	20138
map03420	Nucleotide excision repair	2.79E-09	2.12E-08	27	572	318	20138
map00052	Galactose metabolism	8.71E-09	6.21E-08	18	213	318	20138
map04626	Plant-pathogen interaction	2.46E-08	1.65E-07	34	859	318	20138
map00600	Sphingolipid metabolism	2.82E-06	1.69E-05	13	168	318	20138
map00511	Other glycan degradation	4.71E-06	2.68E-05	13	176	318	20138
map00966	Glucosinolate biosynthesis	1.63E-05	8.86E-05	6	35	318	20138
map00380	Tryptophan metabolism	4.47E-05	0.000232	10	131	318	20138
map00500	Starch and sucrose metabolism	0.000217	0.001078	28	890	318	20138
map04142	Lysosome	0.001034	0.004913	13	327	318	20138
map04144	Endocytosis	0.004142	0.016282	21	707	318	20138
map00909	Sesquiterpenoid and triterpenoid biosynthesis	0.004687	0.017236	5	69	318	20138

Note: x: gene number with a KEGG term in the list; y: gene number with a KEGG term in total; n: gene number in the list; N: total gene number.

Table S16. The GO term enrichment of positive selected genes in *B.oleracea* (D134).

GO ID	GO Term	GO Class	P value	Adjusted P value	x1	x2	n	N	GO level
GO:0004518	nuclease activity	MF	4.35E-06	0.001341	6	136	74	23646	5
GO:0034470	ncRNA processing	BP	0.000256	0.039356	4	98	74	23646	7

Note: x1: gene number with a GO term in the list; x2: gene number with a GO term in total; n: gene number in the list; N: total gene number.

Table S17. The KEGG Pathway enrichment of positive selected genes in *B.oleracea* (D134).

**Map ID**	**Map Title**	**P value**	**Adjusted P value**	**x**	**y**	**n**	**N**
map00360	Phenylalanine metabolism	0.035789821	0.487796584	2	109	55	20138
map00350	Tyrosine metabolism	0.039448466	0.487796584	2	115	55	20138
map00195	Photosynthesis	0.049855919	0.487796584	2	131	55	20138
map00561	Glycerolipid metabolism	0.059988309	0.527897124	3	327	55	20138

Note: x: gene number with a KEGG term in the list; y: gene number with a KEGG term in total; n: gene number in the list; N: total gene number.

Table S18. Summary of aligned sequences, SNP and Indels in D134, 02-12, and TO1000 genomes.

	D134 to 02-12	D134 to TO1000
One to one syntenic blocks	D134	D134
	263,303,513 (68.39%)	318,544,807 (55.39%)
	02-12	TO1000
	263,435,105 (45.78%)	318,737,431 (65.19%)
SNPs	2,057,052	3,963,977
INDELs	434,689	581,173
Insertions(<100bp)	218,423	292,689
Deletions(<100bp)	216,266	288,484

Table S19. Genome distribution of SNP and Indels between D134 and 02-12 genomes.

**Region**	**SNPs**		**D134/02-12 Insertion**		**D134/02-12 Deletion**	
	Number#	Percent	Number#	Percent	Number#	Percent
Intergenic	1549415	0.753221114	144615	0.662086868	142844	0.66050142
Upstream 5kb	123316	0.059947925	22586	0.103404861	22415	0.103645511
exonic	161046	0.078289708	6099	0.027922884	6248	0.028890348
Intron	104918	0.051004058	23996	0.109860225	23687	0.109527156
UP/DOWN	13306	0.00646848	3435	0.015726366	3481	0.016095919
Downstream 5kb	104750	0.050922388	17444	0.079863384	17461	0.080738535
splice	301	0.000146326	205	0.000938546	130	0.000601112
Total	2057052	1	218423	1	216266	1

The statistics was based on the annotation of D134 genome.

Table S20. Genome distribution of SNP and Indels between D134 and TO1000 genomes.

Region	SNPs		D134/TO1000 Insertion		D134/TO1000 Deletion	
	Number#	Percent	Number#	Percent	Number#	Percent
Intergenic	2569338	0.648171773	159427	0.544697614	156958	0.544078701
Upstream 5kb	347841	0.087750509	40464	0.138249131	39873	0.138215638
exonic	388164	0.097922869	10388	0.035491597	10871	0.037683199
Intron	302484	0.076308213	41964	0.143374025	41487	0.143810402
UP/DOWN	48077	0.012128476	7247	0.02476007	7180	0.024888729
Downstream 5kb	306089	0.077217653	32774	0.11197551	31834	0.110349274
splice	1951	0.000492182	360	0.001229974	280	0.000970591
Total	3963977	1	292689	1	288484	1

The statistics was based on the annotation of D134 genome.

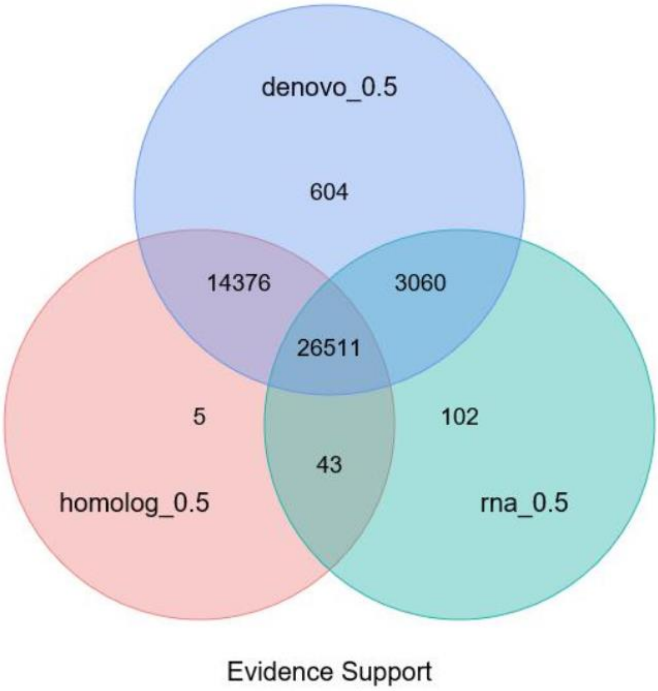

Fig. S1. gene annotation of *B.oleracea* genome. Gene sets generated from the three methods (*De novo* prediction, Homology searching and RNA-seq mapping) are shown.

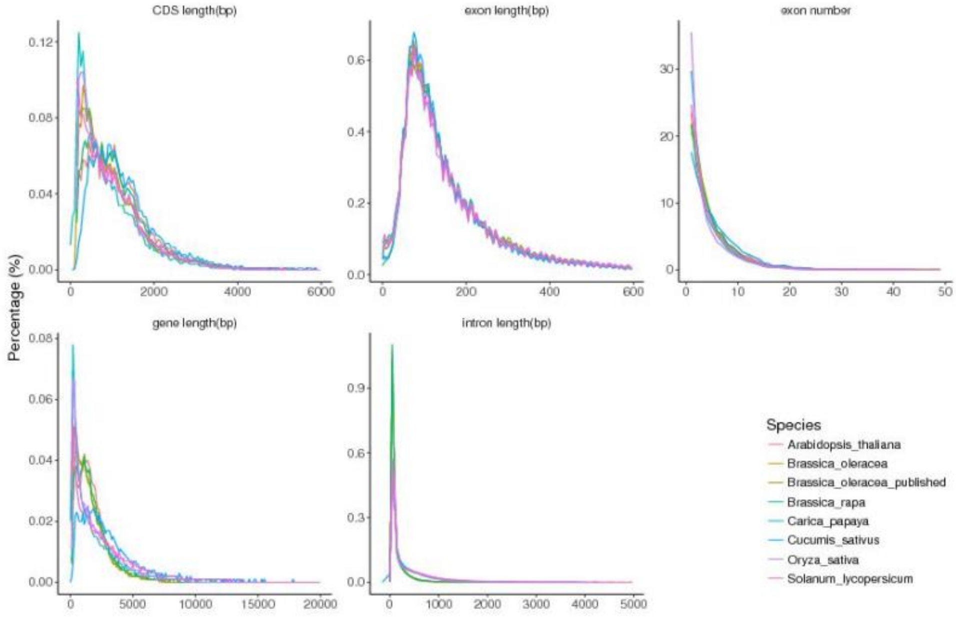

Fig. S2. Gene structure comparision of *B.oleracea* and other six plants. *B. oleracea*, Arabidopsis and *B. rapa* are close ralatives, *B. oleracea* (published) respects 02-12 assembly.

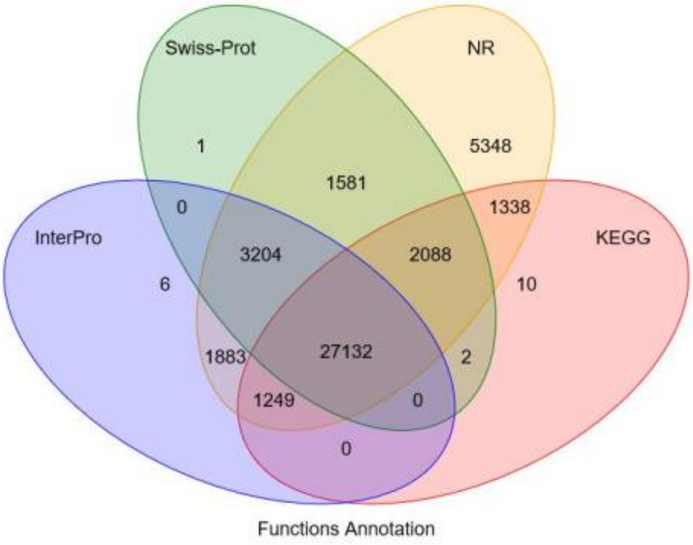

Fig. S3. gene functional annotation of *B.oleracea* genome. Gene sets supported by four databases are shown.

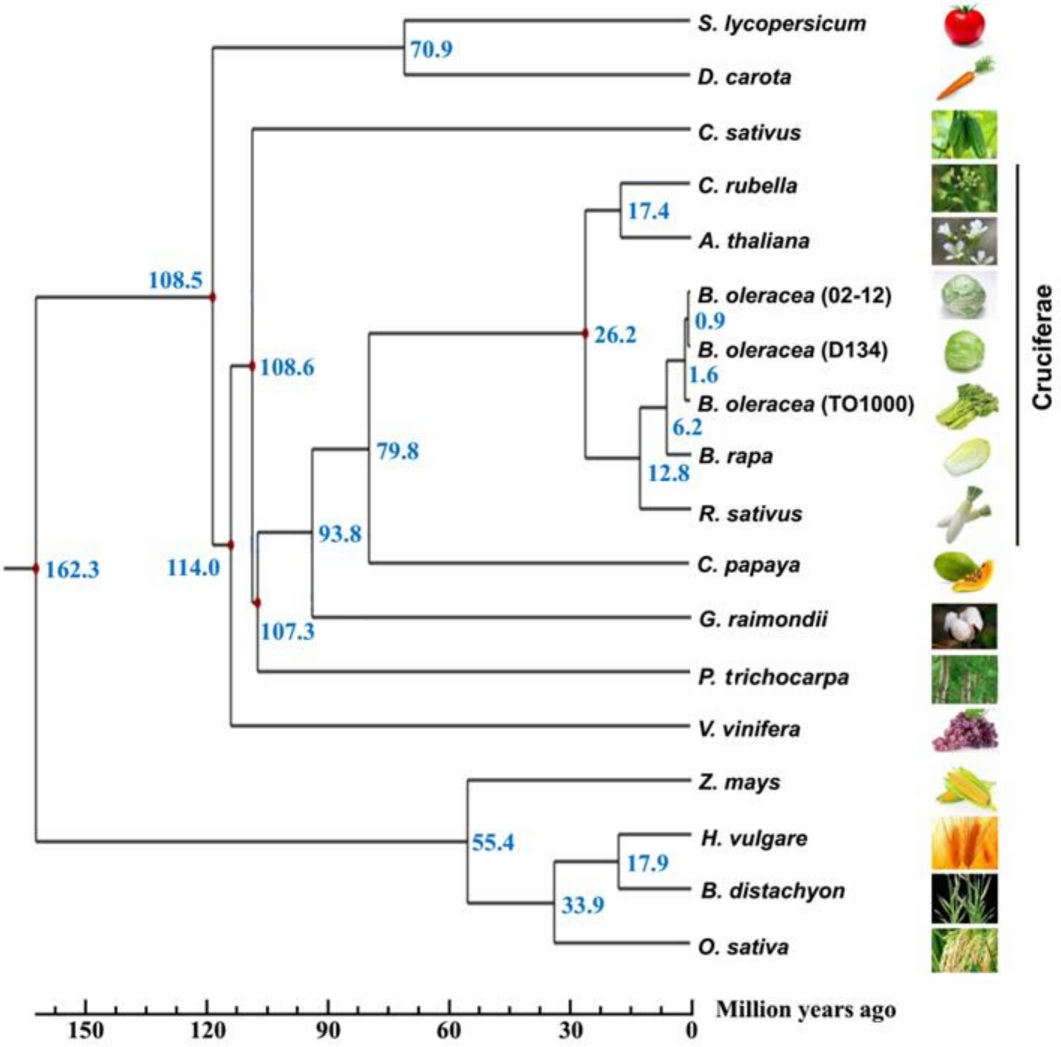

Fig. S4. Phylogenetic tree showing the topology and divergence times for 18 genomes. The phylogenetic tree was constructed based on a concatenated sequence alignment of 432 single-copy gene families from 18 plant species using RAxML software.

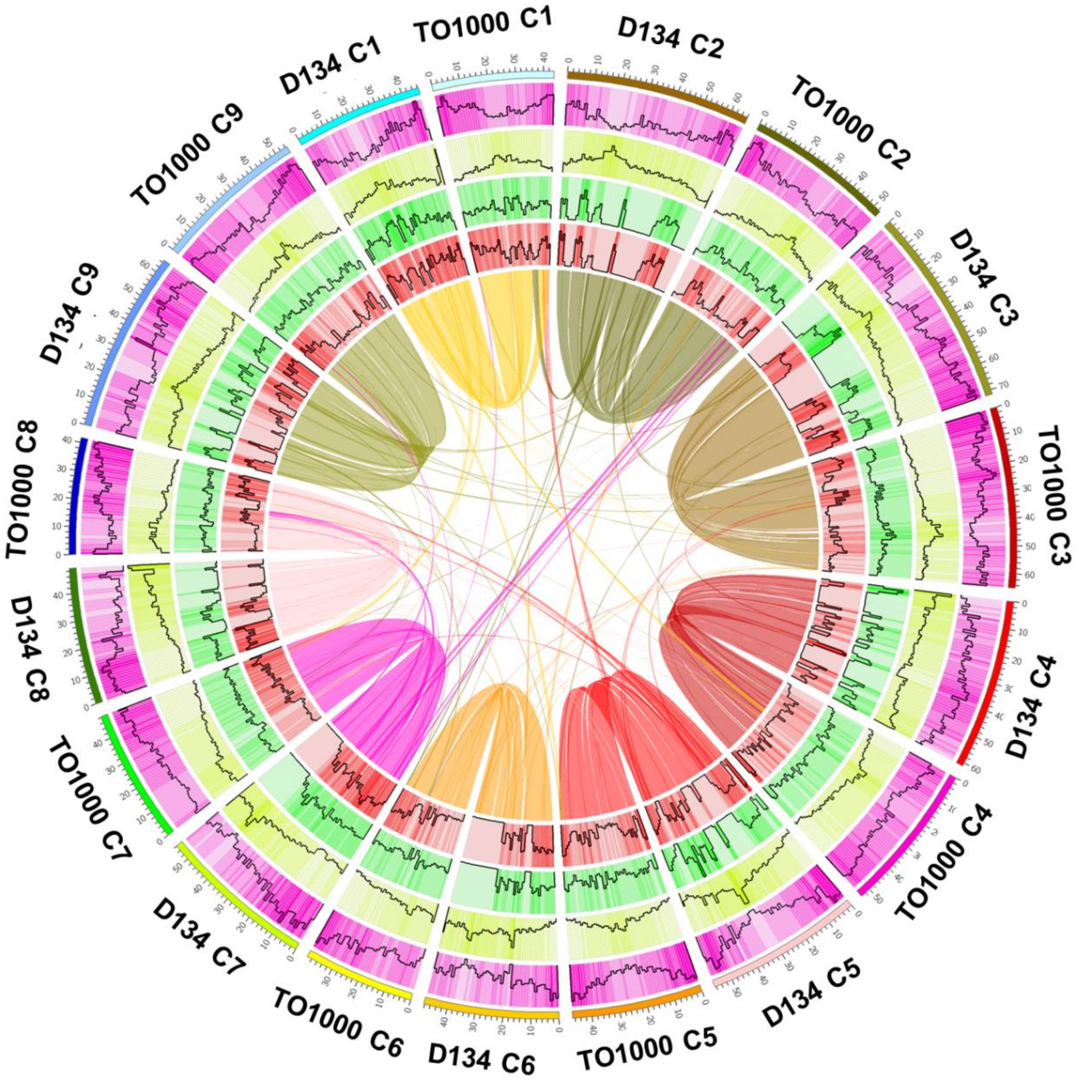

Fig. S5: Genomic landscape of D134 and TO1000.

Chromosomes, gene density, TE density, SNP density, indel density and best-hit gene pairs are in order from outside to inside in the Circos images.
